# Elevated IgG Responses in Infants Are Associated With Reduced Prevalence of *Mycobacterium tuberculosis* Infection

**DOI:** 10.3389/fimmu.2018.01529

**Published:** 2018-07-02

**Authors:** Erin Logan, Angelique Kany Kany Luabeya, Humphrey Mulenga, Dunja Mrdjen, Cynthia Ontong, Adam F. Cunningham, Michele Tameris, Helen McShane, Thomas J. Scriba, William G. C. Horsnell, Mark Hatherill

**Affiliations:** ^1^Division of Immunology, Institute of Infectious Disease and Molecular Medicine, University of Cape Town, Cape Town, South Africa; ^2^South African Tuberculosis Vaccine Initiative (SATVI), Department of Pathology, Institute of Infectious Disease, Molecular Medicine and University of Cape Town, Cape Town, South Africa; ^3^Institutes of Immunology and Immunotherapy and Microbiology and Infection, University of Birmingham, Birmingham, United Kingdom; ^4^The Jenner Institute, University of Oxford, Oxford, United Kingdom; ^5^Laboratory of Molecular and Experimental Immunology and Neurogenetics, UMR 7355, Le Studium Institute for Advanced Studies, CNRS-University of Orléans, Orléans, France

**Keywords:** *Mycobacterium tuberculosis* infection, antibody, enzyme-linked immunosorbent assay, Bacille Calmette-Guérin, vaccine, helminth

## Abstract

**Background:**

It is unclear whether antibodies can prevent *Mycobacterium tuberculosis* (*Mtb*) infection. In this study, we examined the relationship between total plasma IgG levels, IgG elicited by childhood vaccines and soil-transmitted helminths, and *Mtb* infection prevalence, defined by positive QuantiFERON (QFT) test.

**Methods:**

We studied 100 *Mtb* uninfected infants, aged 4–6 months. Ten infants (10%) converted to positive QFT test (QFT+) within 2 years of follow-up for *Mtb* infection. Antibody responses in plasma samples acquired at baseline and tuberculosis investigation were analyzed by enzyme-linked immunosorbent assay and ImmunoCAP^®^ assay.

**Results:**

QFT− infants displayed a significant increase in total IgG titers when re-tested, compared to IgG titers at baseline, which was not observed in QFT+ infants. Bacille Calmette-Guérin (BCG) vaccine-specific IgG2 and live-attenuated measles vaccine-specific IgG were raised in QFT− infants, and infants who acquired an *Mtb* infection did not appear to launch a BCG-specific IgG2 response. IgG titers against the endemic helminth *Ascaris lumbricoides* increased from baseline to QFT re-testing in all infants.

**Conclusion:**

These data show raised IgG associates with a QFT-status. Importantly, this effect was also associated with a trend showing raised IgG titers to BCG and measles vaccine. Our data suggest a possible protective association between raised antibody titers and acquisition of *Mtb* infection, potentially mediated by exposure to antigens both related and unrelated to *Mtb*.

## Introduction

*Mycobacterium tuberculosis* (*Mtb*) infection and tuberculosis (TB) disease represents one of the greatest global infectious disease burdens (1). The only licensed TB vaccine, Bacille Calmette-Guérin (BCG), may partially protect against *Mtb* infection and provides protection against disseminated forms of active TB disease (2–5), but protection against pulmonary TB disease varies widely with age and mycobacterial exposure (6). Development of a vaccine that is more effective than BCG is crucial for the ultimate control of both *Mtb* infection and TB disease (7, 8).

Protection conferred by most clinically efficacious vaccines against their target pathogens requires a protective antibody response (9–11). Antibody responses against *Mtb* do correlate with, and may provide protection against, TB disease (12–17). However, there is no clinical evidence to support the hypothesis that an effective TB vaccine might be based on induction of an antibody response that controls *Mtb* infection.

Our current understanding of how antibodies could protect against *Mtb* infection or TB disease is limited, when compared with our understanding of, for example, T cell-driven immunity to *Mtb*. However, classical functions of antibody such as neutralization, opsonization, and antibody-dependent cellular cytotoxicity (ADCC) have all been demonstrated to contribute to protective immunity against *Mtb*. Antibody opsonization can enhance *Mtb* uptake by (for example) macrophages (12–14), while both antibody neutralization (12, 13) and ADCC-mediated control of *Mtb* have been shown to inhibit bacterial growth (18). The intrinsic features of an antibody also contribute to their ability to control *Mtb*. For example, variation in immunoglobulin glycosylation can influence antibody-mediated control of bacterial replication (18). Different roles for antibody classes in the control of *Mtb* infection have also been demonstrated. In active TB disease, IgG has been shown to impair control and IgA enhance control of disease (19). Therefore, the full range of antibody functions can influence host immunity to *Mtb*, but whether these effects enhance or impede control of infection is likely to vary, depending on whether the host is responding to a latent infection or pathogenic disease.

Any antibody-mediated protection against *Mtb* may also be influenced by host vaccination status and/or history of co-infections unrelated to *Mtb*. Childhood vaccinations, such as live attenuated measles, have been suggested to alter *Mtb*-associated immune responses (20, 21). Additionally, in Sub-Saharan Africa, the potential effect of common endemic infections, such as helminths, on immune responses to concurrent or subsequent *Mtb* infection is an important consideration due to the substantial overlap in prevalence of these diseases (22). Clear evidence exists that helminths can alter host immunity to *Mtb* (23, 24). However, our overall understanding of the influence of bystander antigen/pathogen challenges and immunity to *Mtb* remains unclear, warranting further investigation.

Our study participants live in a region endemic for both TB and soil-transmitted helminths (STH) (25). They receive routine Expanded Programme on Immunization (EPI) vaccinations, which provides a platform to investigate the potential interplay between vaccines, helminth exposure, and *Mtb* immunity. In this study, we addressed whether plasma antibody levels associate with risk of acquiring *Mtb* infection in infants. Additionally, we sought to identify if associations existed between exposure to both related and unrelated antigens/pathogens and altered risk of *Mtb* infection.

## Materials and Methods

### Study Participants

**Figure 1:**
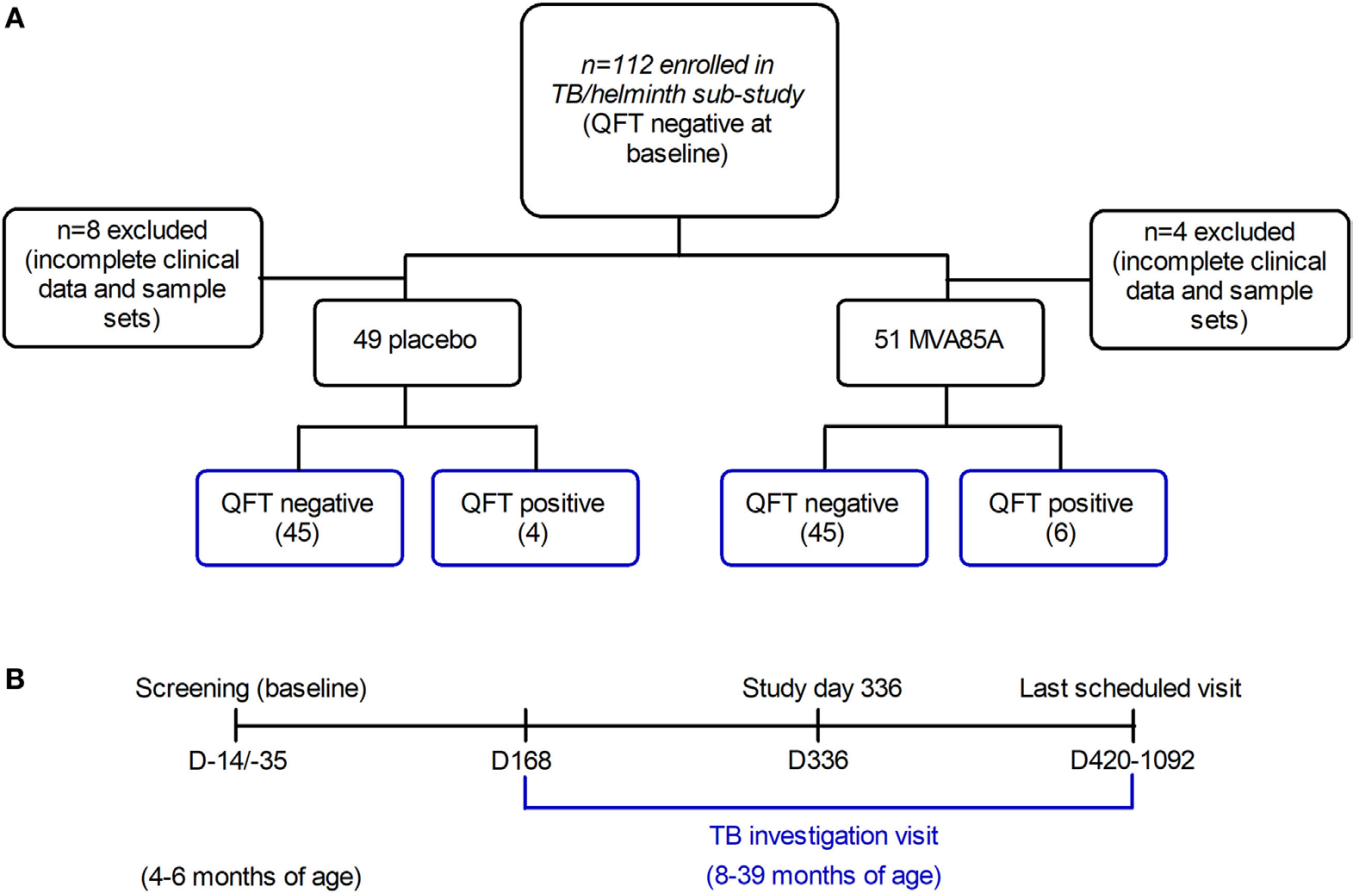
Infants recruited to the tuberculosis (TB)/helminth study. Infants enrolled in the MVA85A trial were randomly assigned to the placebo or MVA85A vaccination arms **(A)**. 112 infants were recruited to the TB/helminth study; incomplete clinical data and sample sets were available for *n* = 12 infants, leaving *n* = 100 available for further analysis. Time-points of blood collection for QFT (QuantiFERON) **(B)** were at baseline, study day 336, at last scheduled visit and/or upon TB investigation. Per participant, serum was analyzed at baseline and upon TB investigation. The age range of infants at baseline was 4–6 months, and the age range at TB investigation was 8–39 months.

This is a sub-study of a double-blind, placebo-controlled efficacy trial of the MVA85A vaccine candidate, performed in the Western Cape Province of South Africa, and which has been described previously (7). Infants aged 4–6 months, who had received BCG vaccine within 7 days of birth, were enrolled. Inclusion criteria at baseline were a negative HIV enzyme-linked immunosorbent assay (ELISA) result; negative QuantiFERON-TB Gold In-tube (QFT) test (used to detect *Mtb* infection by measuring IFNγ responses to specific mycobacterial proteins); and no known exposure to an adult with active TB disease. Infants were expected to have received all routine vaccinations, per the EPI schedule. The trial showed that MVA85A boost vaccination did not provide additional protection against *Mtb* infection or TB disease, compared to BCG vaccination alone in newborns (7). Therefore, this sub-study combines infants from the placebo and MVA85A arms in the same analysis. 112 infants were sequentially recruited into the sub-study at the time of TB investigation (Figure 1A). However, complete clinical data and sample sets were not collected for 12 participants, thus leaving clinical data and samples from 100 participants (89.29%) available for analysis in this study (Figure 1A).

The sub-study and the parent trial (C-020-485) were approved by the University of Cape Town Faculty of Health Sciences Human Research Ethics Committee (032/2010 and 291/2008 respectively); and written informed consent was obtained from the infants’ parents or legal guardians prior to participation.

### *Mtb* Infection

Infants were followed up at 3-monthly intervals for symptoms consistent with TB disease, which if detected, triggered admission for standardized investigation, including QFT re-testing. Infants with symptoms of weight loss or failure to thrive would routinely receive empirical anthelmintic therapy, in addition to regular mass deworming campaigns. Information about prior anthelmintic therapy was not collected. Asymptomatic infants were admitted if an individual with TB disease became a new household contact (7). Plasma samples were analyzed at baseline and at admission for TB disease investigation (Figure 1B). One sample was analyzed per participant, per time-point. *Mtb* infection was defined by a single positive QFT test (i.e., interferon-gamma release assay conversion) at the time of TB investigation. Infants who were QFT positive, but did not show evidence of probable or definite TB disease were classified as *Mtb*-infected. None of the infants, irrespective of QFT status, were diagnosed with active TB disease. Infants were stratified by QFT negative (*Mtb-*uninfected), and QFT positive (*Mtb*-infected) status in the subsequent analyses; none of the infants had active TB disease.

### Antigen Preparation for ELISA

#### Vaccine Antigens

Bacille Calmette-Guérin (Danish strain 1331, Statens Serum Institut, Denmark), live-attenuated measles (Rouvax, Sanofi Pasteur), and tetanus toxoid (Tetavax, Sanofi Pasteur) vaccines were reconstituted in 1× phosphate-buffered saline (PBS) to produce antigen stocks (500 µg/ml); aliquots of stocks were stored at −80°C until required.

#### Helminth Antigen

Whole *Ascaris lumbricoides* worms were treated with a 10× penicillin/streptomycin solution in 1× Amphotericin B (Thermo Fisher Scientific), following which they were washed in filter-sterilized (FS) 1× PBS. Worm sections were then homogenized in FS 1× PBS and centrifuged to remove cellular debris. The protein concentration of the soluble fraction was measured and adjusted to a stock concentration. Aliquots of the stock solution were frozen at −80°C until required.

#### Non-Specific and Antigen-Specific ELISA

The coating IgG antibody used for the total IgG ELISAs was used at 1:5,000 (anti-human IgG Fc-specific; Sigma-Aldrich). All vaccine and helminth coating antigens were used at 5 µg/ml for antigen-specific IgG or 10 µg/ml for antigen-specific IgG subtypes. Initial plasma sample dilutions were 1:50 (total IgG) and 1:20 (antigen-specific), followed by serial 1:5 dilutions of the initial dilutions. Secondary IgG (Southern Biotech) was used at 1:1,000, and secondary IgG subtypes (Southern Biotech) were used at 1:500. All secondary antibodies were alkaline phosphatase-linked.

Antibody ELISAs were performed similarly to methods described previously (19, 26). Briefly, Nunc Immuno Maxisorp 96-well plates (Thermo Fisher Scientific) were coated with antibody or antigen and incubated at 37°C for 3 h, following which plates were blocked overnight at 4°C. Sample was added (50 μl/well) and plates were incubated at 4°C overnight. Secondary antibody was added (50 μl/well) after which the reactions were developed using PNP substrate (Sigma Aldrich). Reactions were stopped with 1 M NaOH and plates read on a Versamax 96-well plate reader (Molecular Devices) at 405 nm (492 nm reference filter). Arbitrary antibody responses were determined from sample titration curves (optical density vs. sample dilution) as previously described (27), enabling a relative assessment of antibody levels in serum. According to this analysis, antibody levels in certain samples and for certain antibody types fell below the detection limit and are observed at 0 on the *y*-axes. This does not preclude the possibility of specific antibody responses within those samples, but may require detection with a more sensitive assay.

### ImmunoCAP^®^ Test

The ImmunoCAP^®^ *in vitro* assay was used to detect antigen-specific IgE responses in serum samples and was used according to the manufacturer’s instructions. An *Ascaris suum*-derived antigen that is cross-reactive with *A. lumbricoides* was used to detect antigen-specific IgE. Responses to this antigen are described as *Ascaris* specific.

### Statistical Analysis

Dot plot graphs are represented with the median and interquartile range where appropriate. Antibody responses in matched sample pairs were compared using the Wilcoxon matched-pairs signed rank test; the Mann–Whitney test was utilized for the analysis of unpaired two-group data. Grouped analyses were investigated by the Kruskal–Wallis test, with the Dunn’s multiple comparison posttest used to compare all pairs of columns. Correlations were investigated using the Spearman correlation test. Where appropriate, analyses were two-tailed. Significance was accepted at *p* ≤ 0.05. Analyses of immunological data were performed using the GraphPad Prism software (v. 5.03).

## Results

### Cohort Description

The median age of infants at TB investigation was 20 months (interquartile range 16–25.63 months). Of the 100 infants analyzed, 10 (10%) tested QFT positive upon re-testing (Table 1).

**Table 1:** Infant sociodemographic and clinical characteristics at tuberculosis (TB) investigation.

	*N* = 100
Infant characteristics	
Age—months [median (interquartile range—IQR)]	20 (16–25.63)
Gender (male)	45 (45%)
Weight at admission—kg [median (IQR)]	9.34 (8.26–10.29)
TB symptoms (e.g., weight loss and failure-to-thrive) YES	60 (60%)
Positive QuantiFERON	10 (10%)

### Infants Displayed an Age-Related Increase in Total IgG Titers

**Figure 2:**
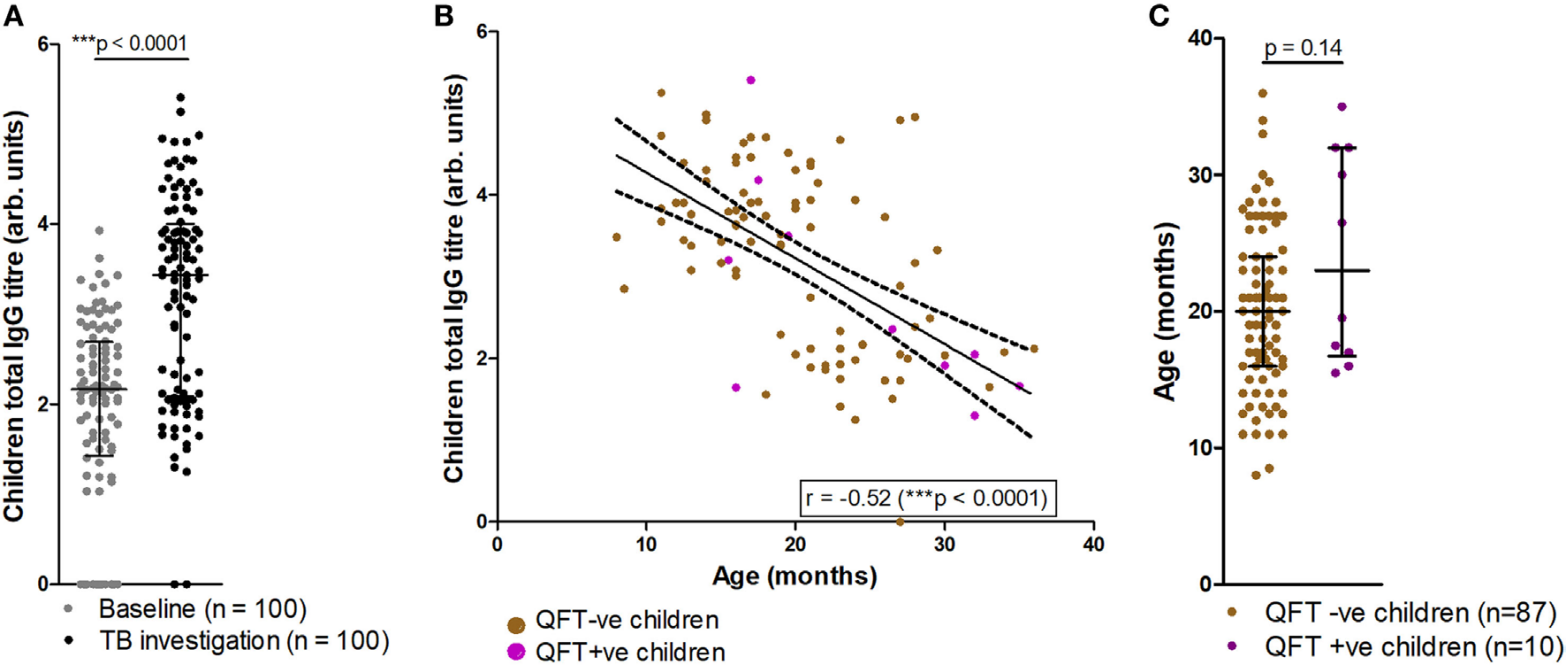
Comparisons of tuberculosis (TB) investigation total IgG responses to baseline total IgG, age and QFT outcome. Total serum IgG in participants at baseline and upon TB investigation visit (*n* = 100) as measured by enzyme-linked immunosorbent assay **(A)**. Total IgG titers vs. age in months at TB investigation (*n* = 97), with samples from QFT positive participants indicated in purple **(B)**; overlaid are the line-of-best-fit and 95% confidence bands (dashed lines). Association between age at TB investigation and QFT result **(C)**. Antibody titers are presented as log-transformed arbitrary values. The Wilcoxon matched-pairs signed rank test was used to assess significance of the comparison in **(A)**, and the Mann–Whitney test was used to assess significance in **(C)**. The Spearman correlation was used to assess the strength of the correlation in **(B)**.

Analysis of total IgG titers revealed a significant increase in total IgG detected at the TB investigation visit, when compared to baseline (Figure 2A). At TB investigation, total IgG titers showed a clear bimodal distribution approximately around the median; further analysis revealed a significant reduction in detected total IgG in older participants (Figure 2B). No significant difference in the median age between QFT− and QFT+ participants was found (median age in months 20 vs. 23, respectively; *p* = 0.14) (Figure 2C).

### Total IgG Titers Increase Significantly in QFT Negative Infants

**Figure 3:**
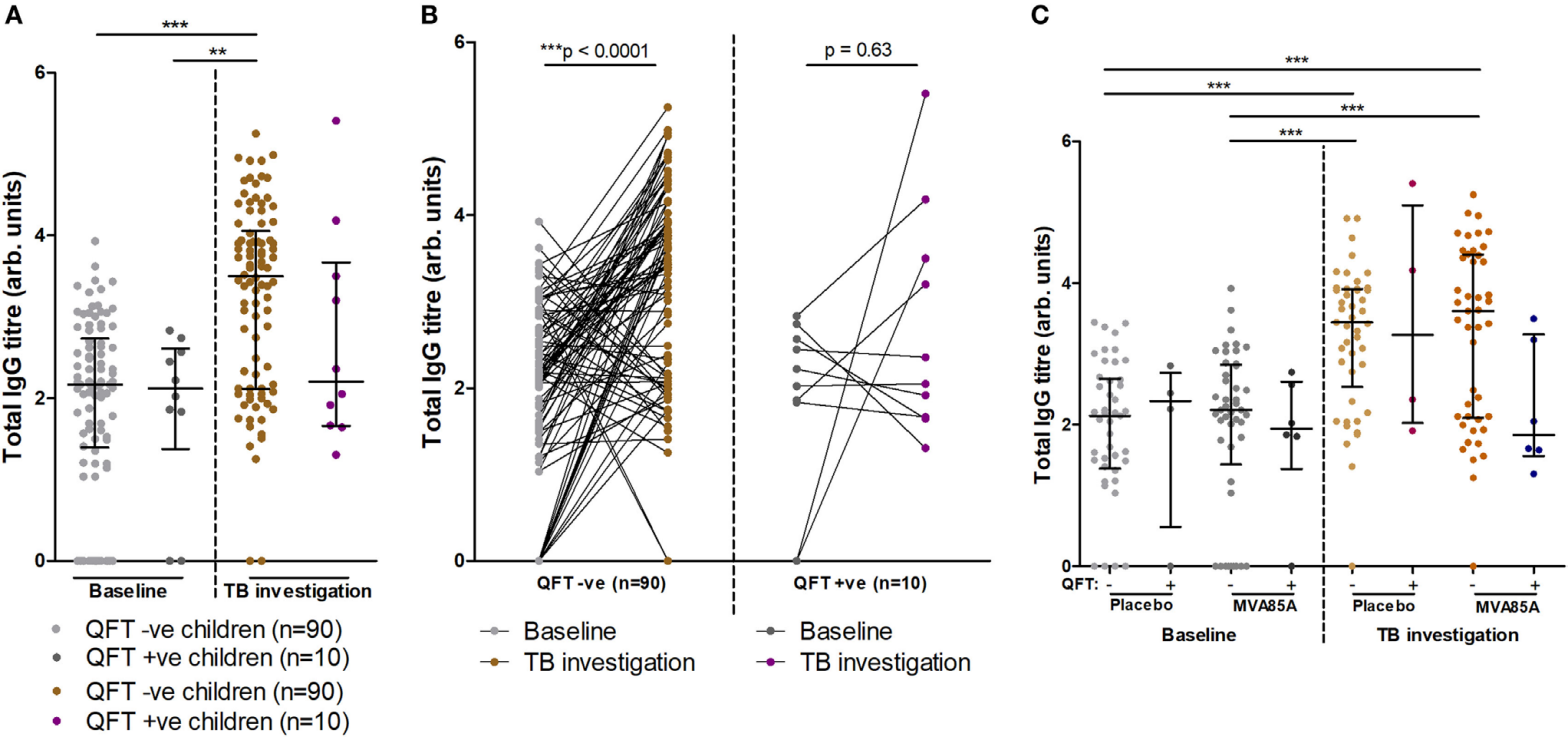
Total IgG titers stratified by QFT outcome and MVA vaccination status. Relationship between QFT outcome and total IgG titers from baseline and tuberculosis (TB) investigation participant samples **(A)**. A before/after comparison of samples subdivided as in **(A)** is presented in **(B)**. Relationship between QFT outcome, MVA85A vaccination status, and total IgG titers from baseline and TB investigation participant samples **(C)**. Column sample numbers left-right **(C)**: 45, 4, 45, 6, 45, 4, 45, 6. Antibody titers are presented as arbitrary values. Statistical analysis was performed with the Kruskal–Wallis test and Dunn’s *post hoc* test to assess significance of the comparisons in **(A,C)**. The Wilcoxon matched-pairs signed rank test was used to assess significance of the associations in **(B)**.

At baseline, infants who did or did not progress to *Mtb* infection had similar total IgG titers (Figure 3A). Analysis of total IgG responses at TB investigation revealed that infants who remained QFT− showed significant increases in total IgG titers from baseline compared to infants who became *Mtb-*infected (QFT+) (Figure 3A). However, there was no significant difference in total IgG titers between the QFT− and QFT+ groups at TB investigation (Figure 3A).

Furthermore, longitudinal analysis of infant IgG responses at baseline and at TB investigation showed significantly increased total IgG titers compared to baseline in infants who remained QFT− on the level of individual participants (Figure 3B). Infants who were QFT+ at TB investigation did not demonstrate significant increases in total IgG (Figure 3B).

The potential influence on total IgG responses of vaccination with MVA85A or placebo (Candin^®^) was also addressed. In agreement with data presented in Figure 3A, we found no significant differences in total IgG between placebo and MVA85A vaccinated study participants at baseline or TB investigation. Moreover, no differences were found in IgG titers between QFT− or QFT+ placebo and MVA85A vaccinated infants (Figure 3C).

### QFT− Status and Vaccine-Specific IgG titers After BCG and Measles Vaccination

**Figure 4:**
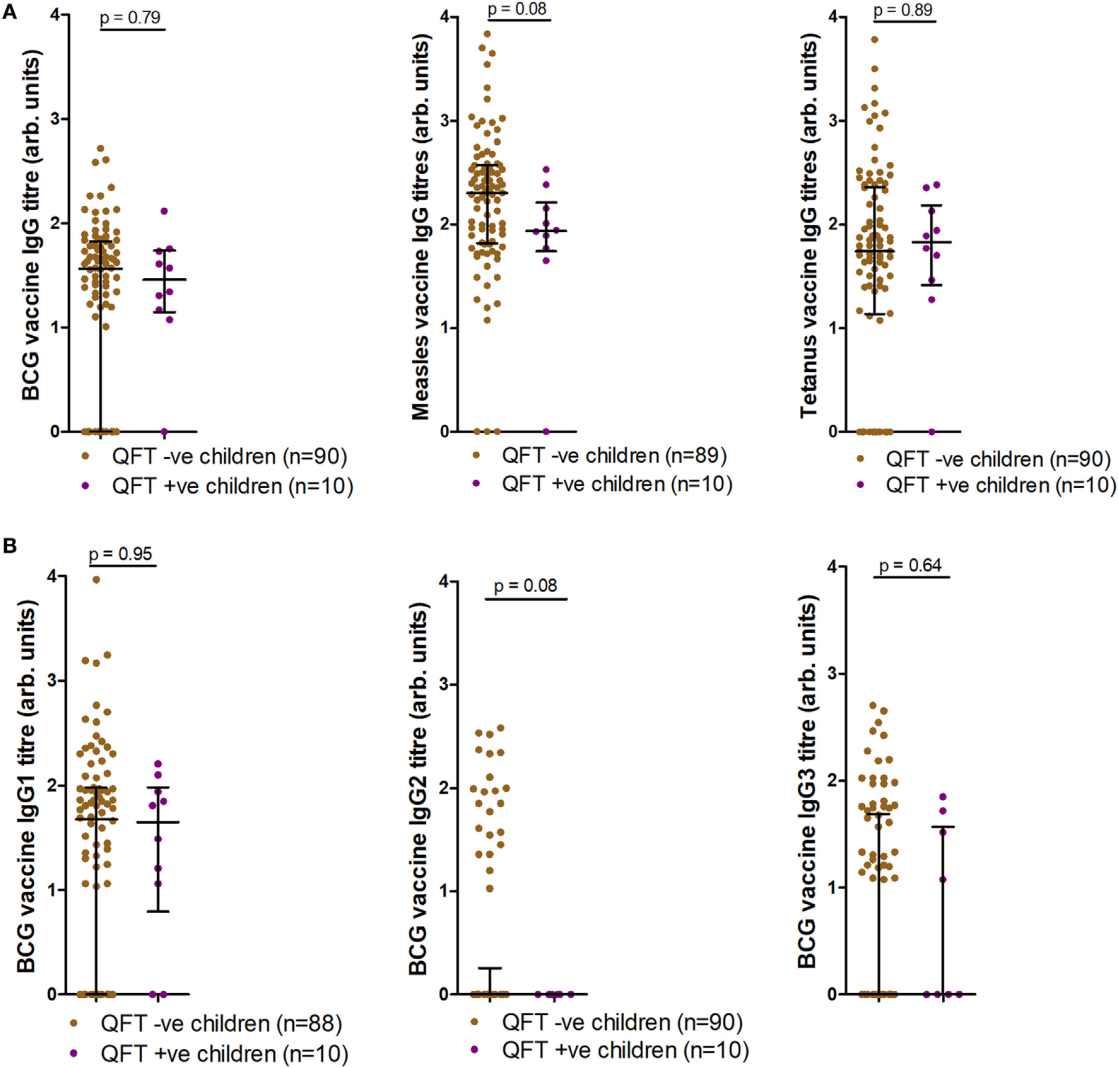
Antibody responses at tuberculosis investigation to childhood vaccines. Anti-Bacille Calmette-Guérin (BCG), measles, and tetanus IgG titers **(A)**, as well as anti-BCG IgG1, IgG2, and IgG3 titers **(B)** as measured by enzyme-linked immunosorbent assay and stratified by QFT result. One sample was excluded in the anti-measles IgG analysis **(A)** (participants had not received measles vaccination). Two fewer samples reported for BCG IgG1 **(B)** due to a lack of sample availability. Antibody titers are presented as arbitrary values. Comparisons were assessed for significance by the Mann–Whitney test.

We next tested whether QFT status associated with antibody responses to BCG or to heterologous antigens. Antigen-specific antibody responses to BCG, live-attenuated measles, and tetanus toxoid vaccines were also measured at TB investigation and compared to infant *Mtb* infection outcome. Analysis of IgG responses to BCG, measles, and tetanus toxoid revealed a trend for increased measles-specific IgG titers (*p* = 0.08) in infants who did not acquire *Mtb* infection, but no association between BCG or tetanus toxoid IgG and *Mtb* infection outcome (Figure 4A). Analysis of BCG IgG subtype-specific responses revealed a trend for increased BCG-specific IgG2 titers (*p* = 0.08) in infants who did not acquire *Mtb* infection, but no association between BCG-specific IgG1 or IgG3 and *Mtb* infection outcome (Figure 4B). This finding was related to significant inverse correlations between measles-specific IgG and quantitative QFT values, and between BCG-specific IgG2 and quantitative QFT values (Figures S1A,B in Supplementary Material). No associations were found with BCG-specific IgG/IgG1/IgG3 or tetanus-specific IgG titers and quantitative QFT values (Figures S1C–F in Supplementary Material). However, it is important to note that all participants who had *Mtb* infection but not disease had low levels of detectable IFNγ. As expected (28), tetanus toxoid-specific IgG titers significantly correlated with age at follow-up (Figure S2F in Supplementary Material). No relationship with age was found with anti-BCG or live-attenuated measles IgG titers at follow-up (Figure S2 in Supplementary Material).

### Soil-Transmitted Helminth-Specific IgG Titers Were Similar in QFT− and QFT+ Infants

**Figure 5:**
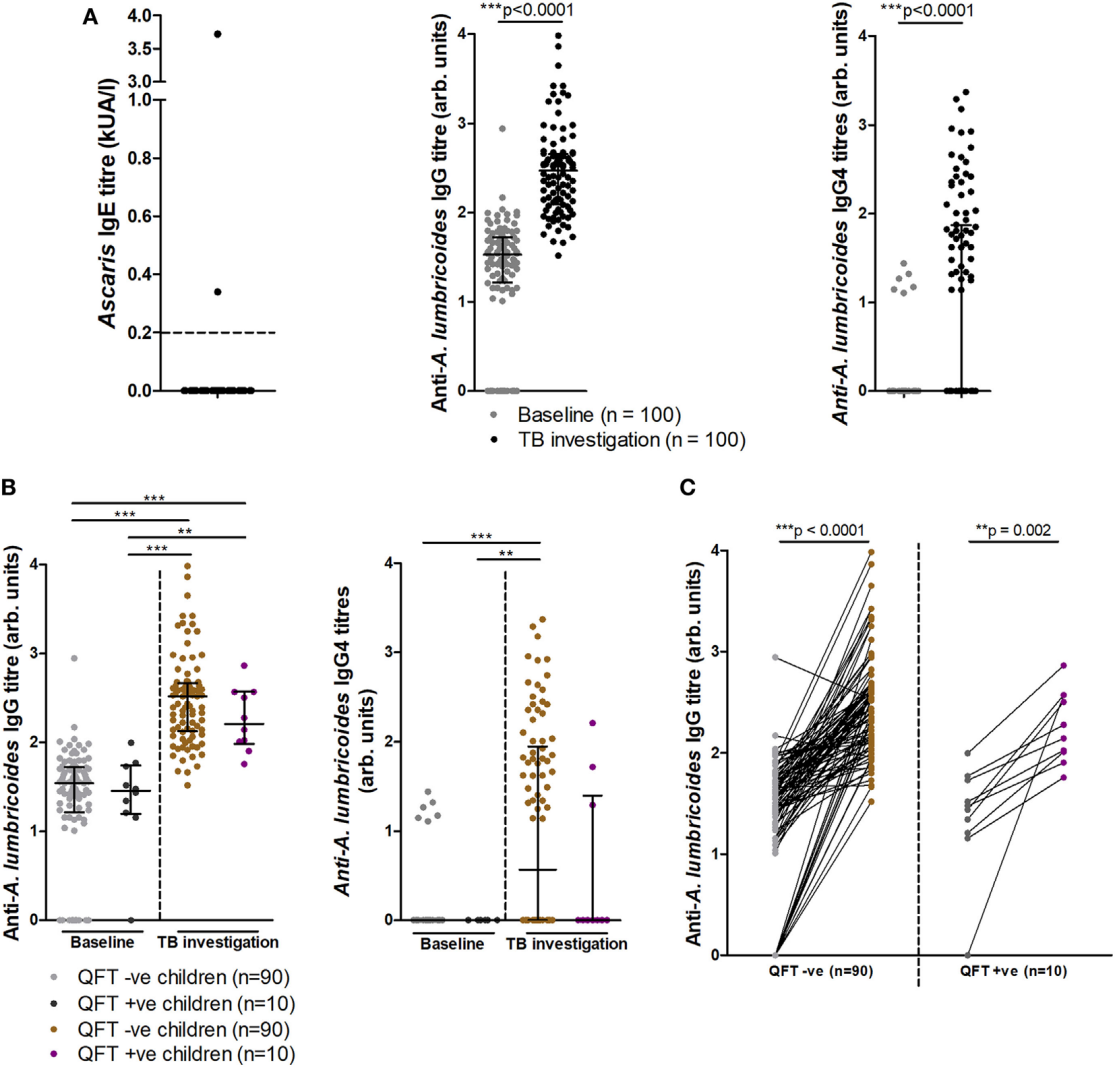
Soil-transmitted helminth-specific IgG titers and QFT outcome at baseline and upon tuberculosis investigation. Anti-*Ascaris lumbricoides* IgE (*n* = 50), IgG, and IgG4 (*n* = 100) titers **(A)**. Anti-*A. lumbricoides* IgG and IgG4 titers stratified by QFT outcome **(B)**. A before/after comparison of anti-*A. lumbricoides* IgG titers as subdivided in **(B)** is presented in **(C)**. Antibody titers are presented as arbitrary values. The Mann–Whitney test was used to assess two-group comparisons for significance in **(A)**, the Kruskal–Wallis test with Dunn’s *post hoc* test was used for the multi-group comparisons in **(B)**, and the Wilcoxon matched-pairs signed rank test was used to assess for significance in **(C)**.

Since helminth exposure can alter risk and outcome of *Mtb* disease (29–31), we examined if any relationship existed between infant exposure to *A. lumbricoides* and *Mtb* infection. Of 91 stool samples obtained, none tested positive for active helminth infection (Table S1 in Supplementary Material). However, anti-*Ascaris* IgE and IgG, indicative of prior exposure to the parasite, were found in infants (Figure 5A). At TB investigation, 2/50 (4%) infants tested for IgE responses presented with raised *Ascaris*-specific IgE, as detected by ImmunoCAP^®^ (Figure 5A). Additionally, 85/100 (85%) infants at baseline and all infants at TB investigation had detectable anti-*A. lumbricoides* IgG, with these levels increasing significantly from baseline to TB investigation (Figure 5A). Analysis of IgG subtype responses revealed that 6/100 (6%) infants at baseline and 48/100 (48%) infants at TB investigation had detectable levels of anti-*A. lumbricoides* IgG4 (Figure 5A). Anti-*A. lumbricoides* IgG nor IgG4 titers were not significantly different between QFT− infants and QFT+ infants (Figure 5B).

Longitudinal analysis of individual infant anti-*A. lumbricoides* IgG responses at baseline and TB investigation showed significantly increased total IgG titers in both QFT− and QFT+ infants, indicating an increase in anti-*A. lumbricoides* IgG responses over time (Figure 5C). Others have suggested that helminth infection negatively influences the magnitude of the QFT IFNγ response, resulting in a higher likelihood of an indeterminate result (32, 33). Although no association was found between anti-*A. lumbricoides* IgG and quantitative IFNγ in the same samples (as detected by QFT), a significant inverse correlation was found between anti-*A. lumbricoides* IgG4 and quantitative IFNγ levels (Figure S3 in Supplementary Material).

## Discussion

In this study, we found that infants who did not acquire *Mtb* infection had significantly increased total IgG titers from baseline to TB investigation, unlike infants who became *Mtb* infected. This effect was related to a trend showing raised IgG titers against BCG and measles vaccination. Together, these findings suggest that raised specific and heterologous IgG titers may play a role in host responses that protect against *Mtb* infection. Therefore, induction of protective IgG responses in QFT− infants may be a feature of immune activation by both pathogen-related and unrelated antigens.

Initial findings indicate that total IgG responses increase significantly from baseline to TB investigation; however, at TB investigation, there was an inverse correlation between total IgG titers and age. It is important to note that the total IgG titers presented are from two distinct age ranges, namely 4–6 months at baseline and 8–39 months at TB investigation, and that the range of antibody titer values is wide. Additionally, older infants tend to fall within the lower half of the bimodal sample distribution observed at TB investigation, indicating why an inverse correlation between age and antibody titer is possible despite a higher median total IgG titer at TB investigation than at baseline.

Our findings build on recent reports demonstrating that antibody responses differentially contribute to control of *Mtb* infection and disease. Studies have addressed *Mtb* antigen-specific immunoglobulin responses as contributing to a range of classical antibody-mediated responses (18, 19). In this study, we observed that only QFT− infants showed a significant increase in total IgG titers from baseline to TB investigation, suggesting an association between raised IgG and reduced risk of *Mtb* infection, highlighting the potential importance of antibodies in protective responses against *Mtb*.

It has long been known that BCG vaccination stimulates *Mycobacterium*-specific antibody production (34–36). Induction of IgM and IgG has been observed, with IgG1, IgG2, and IgG3 as the most prevalent IgG subtypes detected (37, 38). Our identification of BCG-induced antibody responses in this study supports these previous studies. Our findings also suggest that infants who became *Mtb* infected did not launch a BCG-specific IgG2 response, which could indicate some impairment of the humoral response to BCG vaccination in these infants, which in turn may be implicated in increased susceptibility to *Mtb* infection. However, the current study lacks sufficient participant numbers and power to address this question adequately and a larger cohort study would be required to analyze the importance of this finding. Nevertheless, to the best of our knowledge, this observation is novel and warrants further investigation.

We also identified a trend toward increased measles vaccine-specific IgG titers in QFT− infants as compared to QFT+ infants. However, since this difference was not statistically significant, the possibility that measles vaccination might provide heterologous protection against acquisition of *Mtb* infection remains speculative. Measles vaccination has previously been shown to decrease responsiveness to the tuberculin skin test in individuals with a positive reaction to purified protein derivative and/or active TB disease (20, 21). Conversely others have suggested that certain childhood vaccines, such as measles (and BCG), may provide protection against all-cause mortality (39, 40).

We acknowledge that generalizability of the abovementioned results is limited by the sample size and that other infections at the time of TB investigation may have contributed to total IgG titers. However, it is unlikely that other infections would contribute differentially to IgG titers in QFT+ vs. QFT− children, or to BCG-specific or measles vaccine-specific antibody titers.

We found no association between anti-*A. lumbricoides* IgG or IgG4 responses and *Mtb* infection, suggesting that anti-helminth responses do not underlie the differences observed with the total IgG responses in this cohort. Our current understanding of how helminth infection/exposure may influence *Mtb* infection is incomplete. Rodent studies have presented evidence of positive (23), neutral (41–43) and negative effects (30, 44) on host control of *Mtb* infection by helminths. Clinical reports of the relationship between helminth and *Mtb* infections are also not consistent, which is likely to reflect the diverse contexts of both helminth or *Mtb* infection/disease. Similar studies to ours also did not identify positive or negative associations between helminth exposure and *Mtb* infection or BCG efficacy (45–47).

We found that few infants had raised anti-*A. lumbricoides* IgE, in agreement with reports by others that anti-*Ascaris* IgE is primarily detected in older children and IgG4 is primarily detected in younger children (48). We propose that the raised levels of anti-*A. lumbricoides* IgG we observed are reflective of prior exposure to helminths. The unexpectedly low levels of current infection (25) may be attributable to appropriate household sanitation, water provision (Table S1 in Supplementary Material), and mass de-worming campaigns in the study community. Regardless, infants in this study showed evidence of immunological memory to helminth infection.

This study did not address antibody responses to *Mtb*-specific antigens due to a lack of antigen availability, and limited samples could not be used to optimize functional assays for more in-depth analysis of antibody responses. It is also important to consider the impact that small sample size may have had on the findings presented here. Despite a 2-year cumulative incidence of *Mtb* infection of 10%, the number of infants in the study who demonstrated QFT conversion was small. A larger sample size would be required to investigate these initial findings more thoroughly. Notably, antigen-specific antibody responses and their association with *Mtb* infection and/or TB disease have been investigated in a similar cohort; here, antigen 85A-specfic IgG responses were associated with a difference in the occurrence of TB disease, but not *Mtb* infection (15). However, a direct comparison between studies would be inaccurate due to the difference in antigen specificity and design of the assays, as well as the discrepancy in sample size between studies.

The data presented here suggest that both pathogen related and heterologous immune activation of the immature immune system by childhood vaccination may be implicated in reduced risk of acquiring an *Mtb* infection. The central finding that infants who did not acquire *Mtb* infection exhibited significantly increased total IgG titers from baseline to the time of TB investigation warrants further investigation of the specific components of the total IgG response responsible for this observation.

## Ethics Statement

This study was carried out in accordance with the recommendations of the University of Cape Town Faculty of Health Sciences Human Research Ethics Committee. The protocol was approved by the University of Cape Town Faculty of Health Sciences Human Research Ethics Committee. Written informed consent was obtained from the infants’ parents or legal guardians in accordance with the Declaration of Helsinki.

## Author Contributions

AL, WH, and MH contributed to conceptualization and design of the study; EL performed experiments and data analysis; AL was the site investigator of the helminth study and analyzed clinical and sociodemographic data; MT and MH were principal investigators of the parent trial; HM was the study data manager; CO was the study coordinator; EL, TS, WH, and MH drafted the manuscript; EL, AL, DM, AC, MT, HS, WH, and MH contributed to manuscript revision and critical analysis.

## Conflict of Interest Statement

The authors declare that the research was conducted in the absence of any commercial or financial relationships that could be construed as a potential conflict of interest.

## References

[1] WHO. Global Tuberculosis Report. Geneva: World Health Organization (2016).

[2] Fine PE Variation in protection by BCG: implications of and for heterologous immunity. Lancet (1995) 346(8986):1339–45. 10.1016/S0140-6736(95)92348-9 7475776

[3] Colditz GA Berkey CS Mosteller F Brewer TF Wilson ME Burdick E The efficacy of bacillus Calmette-Guerin vaccination of newborns and infants in the prevention of tuberculosis: meta-analyses of the published literature. Pediatrics (1995) 96(1 Pt 1):29–35. 7596718

[4] Roy A Eisenhut M Harris RJ Rodrigues LC Sridhar S Habermann S Effect of BCG vaccination against *Mycobacterium tuberculosis* infection in children: systematic review and meta-analysis. BMJ (2014) 349:g4643. 10.1136/bmj.g4643 25097193 PMC4122754

[5] Basu Roy R Sotgiu G Altet-Gomez N Tsolia M Ruga E Velizarova S Identifying predictors of interferon-gamma release assay results in pediatric latent tuberculosis: a protective role of bacillus Calmette-Guerin? A pTB-NET collaborative study. Am J Respir Crit Care Med (2012) 186:378–84. 10.1164/rccm.201201-0026OC 22700862 PMC3443812

[6] Mangtani P Abubakar I Ariti C Beynon R Pimpin L Fine PE Protection by BCG vaccine against tuberculosis: a systematic review of randomized controlled trials. Clin Infect Dis (2014) 58(4):470–80. 10.1093/cid/cit790 24336911

[7] Tameris MD Hatherill M Landry BS Scriba TJ Snowden MA Lockhart S Safety and efficacy of MVA85A, a new tuberculosis vaccine, in infants previously vaccinated with BCG: a randomised, placebo-controlled phase 2b trial. Lancet (2013) 381(9871):1021–8. 10.1016/S0140-6736(13)60177-4 23391465 PMC5424647

[8] Andersen P Kaufmann SH Novel vaccination strategies against tuberculosis. Cold Spring Harb Perspect Med (2014) 4(6):1–20. 10.1101/cshperspect.a018523 PMC4031959 24890836

[9] Achkar JM Casadevall A. Antibody-mediated immunity against tuberculosis: implications for vaccine development. Cell Host Microbe (2013) 13(3):250–62. 10.1016/j.chom.2013.02.009 23498951 PMC3759397

[10] Siegrist C-A Vaccine immunology. Vaccines (Basel) (2008) 5:1725.

[11] Robbins JB Schneerson R Szu SC. Hypothesis: how licensed vaccines confer protective immunity. Adv Exp Med Biol (1996) 397:169–82. 10.1007/978-1-4899-1382-1_22 8718596

[12] de Valliere S Abate G Blazevic A Heuertz RM Hoft DF. Enhancement of innate and cell-mediated immunity by antimycobacterial antibodies. Infect Immun (2005) 73(10):6711–20. 10.1128/IAI.73.10.6711-6720.2005 16177348 PMC1230956

[13] Chen T Blanc C Eder AZ Prados-Rosales R Souza AC Kim RS Association of human antibodies to arabinomannan with enhanced mycobacterial opsonophagocytosis and intracellular growth reduction. J Infect Dis (2016) 214(2):300–10. 10.1093/infdis/jiw141 27056953 PMC4918826

[14] Kumar SK Singh P Sinha S. Naturally produced opsonizing antibodies restrict the survival of *Mycobacterium tuberculosis* in human macrophages by augmenting phagosome maturation. Open Biol (2015) 5(12):150171. 10.1098/rsob.150171 26674415 PMC4703058

[15] Fletcher HA Snowden MA Landry B Rida W Satti I Harris SA T-cell activation is an immune correlate of risk in BCG vaccinated infants. Nat Commun (2016) 7:11290. 10.1038/ncomms11290 27068708 PMC4832066

[16] Maglione PJ Xu J Casadevall A Chan J. Fc gamma receptors regulate immune activation and susceptibility during *Mycobacterium tuberculosis* infection. J Immunol (2008) 180(5):3329–38. 10.4049/jimmunol.180.5.3329 18292558

[17] Teitelbaum R Glatman-Freedman A Chen B Robbins JB Unanue E Casadevall A A mAb recognizing a surface antigen of *Mycobacterium tuberculosis* enhances host survival. Proc Natl Acad Sci U S A (1998) 95(26):15688–93. 10.1073/pnas.95.26.15688 9861031 PMC28105

[18] Lu LL Chung AW Rosebrock TR Ghebremichael M Yu WH Grace PS A functional role for antibodies in tuberculosis. Cell (2016) 167(2):433–443.e14. 10.1016/j.cell.2016.08.072 27667685 PMC5526202

[19] Zimmermann N Thormann V Hu B Kohler AB Imai-Matsushima A Locht C Human isotype-dependent inhibitory antibody responses against *Mycobacterium tuberculosis*. EMBO Mol Med (2016) 8(11):1325–39. 10.15252/emmm.201606330 27729388 PMC5090662

[20] Mellman WJ Wetton R Depression of the tuberculin reaction by attenuated measles virus vaccine. J Lab Clin Med (1963) 61:453–8. 13934705

[21] Brody JA Overfield T Hammes LM Depression of the tuberculin reaction by viral vaccines. N Engl J Med (1964) 271:1294–6. 10.1056/NEJM196412172712505 14214636

[22] Hotez PJ Mistry N Rubinstein J Sachs JD Integrating neglected tropical diseases into AIDS, tuberculosis, and malaria control. N Engl J Med (2011) 364(22):2086–9. 10.1056/NEJMp1014637 21631320

[23] du Plessis N Kleynhans L Thiart L van Helden PD Brombacher F Horsnell WG Acute helminth infection enhances early macrophage mediated control of mycobacterial infection. Mucosal Immunol (2013) 6(5):931–41. 10.1038/mi.2012.131 23250274

[24] Elias D Akuffo H Pawlowski A Haile M Schon T Britton S. Schistosoma mansoni infection reduces the protective efficacy of BCG vaccination against virulent *Mycobacterium tuberculosis*. Vaccine (2005) 23(11):1326–34. 10.1016/j.vaccine.2004.09.038 15661380

[25] Hotez PJ Kamath A. Neglected tropical diseases in sub-saharan Africa: review of their prevalence, distribution, and disease burden. PLoS Negl Trop Dis (2009) 3(8):e412. 10.1371/journal.pntd.0000412 19707588 PMC2727001

[26] Cooper PJ Espinel I Wieseman M Paredes W Espinel M Guderian RH Human onchocerciasis and tetanus vaccination: impact on the postvaccination antitetanus antibody response. Infect Immun (1999) 67(11):5951–7. 10531253 10.1128/iai.67.11.5951-5957.1999 PMC96979

[27] Bobat S Darby M Mrdjen D Cook C Logan E Auret J Natural and vaccine-mediated immunity to *Salmonella typhimurium* is impaired by the helminth *Nippostrongylus brasiliensis*. PLoS Negl Trop Dis (2014) 8(12):e3341. 10.1371/journal.pntd.0003341 25474738 PMC4256288

[28] Borrow RBP Roper MH The Immunological Basis for Immunization Series. Module 3: Tetanus Update 2006. Switzerland: WHO (2007).

[29] George PJ Kumar NP Sridhar R Hanna LE Nair D Banurekha VV Coincident helminth infection modulates systemic inflammation and immune activation in active pulmonary tuberculosis. PLoS Negl Trop Dis (2014) 8(11):e3289. 10.1371/journal.pntd.0003289 25375117 PMC4222842

[30] Potian JA Rafi W Bhatt K McBride A Gause WC Salgame P. Preexisting helminth infection induces inhibition of innate pulmonary anti-tuberculosis defense by engaging the IL-4 receptor pathway. J Exp Med (2011) 208(9):1863–74. 10.1084/jem.20091473 21825018 PMC3171086

[31] Perry S Hussain R Parsonnet J. The impact of mucosal infections on acquisition and progression of tuberculosis. Mucosal Immunol (2011) 4(3):246–51. 10.1038/mi.2011.11 21412228 PMC5480373

[32] Lucas M Nicol P McKinnon E Whidborne R Lucas A Thambiran A A prospective large-scale study of methods for the detection of latent *Mycobacterium tuberculosis* infection in refugee children. Thorax (2010) 65(5):442–8. 10.1136/thx.2009.127555 20435869

[33] Thomas TA Mondal D Noor Z Liu L Alam M Haque R Malnutrition and helminth infection affect performance of an interferon gamma-release assay. Pediatrics (2010) 126(6):e1522–9. 10.1542/peds.2010-0885 21059723 PMC3403682

[34] Diena BB Yugi H Wallace R Carriere J Greenberg L The bentonite flocculation test in the serology of tuberculosis. I. Purification of BCG antigens. Can J Microbiol (1968) 14(8):881–5. 10.1139/m68-148 4875429

[35] Bardana EJ Jr McClatchy JK Farr RS Minden P Universal occurrence of antibodies to tubercle bacilli in sera from non-tuberculous and tuberculous individuals. Clin Exp Immunol (1973) 13(1):65–77. 4202948 PMC1553751

[36] Parlett RC Youmans GP An evaluation of the specificity and sensitivity of a gel double-diffusion test for tuberculosis. Am Rev Respir Dis (1959) 80:153–66. 10.1164/arrd.1959.80.2.153 14430545

[37] Hoft DF Kemp EB Marinaro M Cruz O Kiyono H McGhee JR A double-blind, placebo-controlled study of *Mycobacterium*-specific human immune responses induced by intradermal bacille Calmette-Guerin vaccination. J Lab Clin Med (1999) 134(3):244–52. 10.1016/S0022-2143(99)90204-4 10482309

[38] Beyazova U Rota S Cevheroglu C Karsligil T. Humoral immune response in infants after BCG vaccination. Tuber Lung Dis (1995) 76(3):248–53. 10.1016/S0962-8479(05)80013-9 7548909

[39] Goodridge HS Ahmed SS Curtis N Kollmann TR Levy O Netea MG Harnessing the beneficial heterologous effects of vaccination. Nat Rev Immunol (2016) 16(6):392–400. 10.1038/nri.2016.43 27157064 PMC4931283

[40] Higgins JP Soares-Weiser K Lopez-Lopez JA Kakourou A Chaplin K Christensen H Association of BCG, DTP, and measles containing vaccines with childhood mortality: systematic review. BMJ (2016) 355:i5170. 10.1136/bmj.i5170 27737834 PMC5063034

[41] Rafi W Bhatt K Gause WC Salgame P. Neither primary nor memory immunity to *Mycobacterium tuberculosis* infection is compromised in mice with chronic enteric helminth infection. Infect Immun (2015) 83(3):1217–23. 10.1128/IAI.03004-14 25605766 PMC4333454

[42] Hubner MP Killoran KE Rajnik M Wilson S Yim KC Torrero MN Chronic helminth infection does not exacerbate *Mycobacterium tuberculosis* infection. PLoS Negl Trop Dis (2012) 6(12):e1970 10.1371/journal.pntd.0001970 23285308 PMC3529511

[43] Frantz FG Rosada RS Turato WM Peres CM Coelho-Castelo AA Ramos SG The immune response to toxocariasis does not modify susceptibility to *Mycobacterium tuberculosis* infection in BALB/c mice. Am J Trop Med Hyg (2007) 77(4):691–8. 10.4269/ajtmh.2007.77.691 17978073

[44] Monin L Griffiths KL Lam WY Gopal R Kang DD Ahmed M Helminth-induced arginase-1 exacerbates lung inflammation and disease severity in tuberculosis. J Clin Invest (2015) 125(12):4699–713. 10.1172/JCI77378 26571397 PMC4665786

[45] Biraro IA Egesa M Toulza F Levin J Cose S Joloba M Impact of co-infections and BCG immunisation on immune responses among household contacts of tuberculosis patients in a Ugandan cohort. PLoS One (2014) 9(11):e111517. 10.1371/journal.pone.0111517 25372043 PMC4221037

[46] Lule SA Mawa PA Nkurunungi G Nampijja M Kizito D Akello F Factors associated with tuberculosis infection, and with anti-mycobacterial immune responses, among five year olds BCG-immunised at birth in Entebbe, Uganda. Vaccine (2015) 33(6):796–804. 10.1016/j.vaccine.2014.12.015 25529292 PMC4317190

[47] Ndibazza J Mpairwe H Webb EL Mawa PA Nampijja M Muhangi L Impact of anthelminthic treatment in pregnancy and childhood on immunisations, infections and eczema in childhood: a randomised controlled trial. PLoS One (2012) 7(12):e50325. 10.1371/journal.pone.0050325 23236367 PMC3517620

[48] Turner JD Faulkner H Kamgno J Kennedy MW Behnke J Boussinesq M Allergen-specific IgE and IgG4 are markers of resistance and susceptibility in a human intestinal nematode infection. Microbes Infect (2005) 7(7–8):990–6. 10.1016/j.micinf.2005.03.036 15961339

