# Supplementary figures and images for: Elevated IgG Responses in Infants Are Associated With Reduced Prevalence of Mycobacterium tuberculosis Infection
Source: Front Immunol. 2018 Jul 2;9:1529. doi: 10.3389/fimmu.2018.01529 (PMC6036805; doi:10.3389/fimmu.2018.01529)

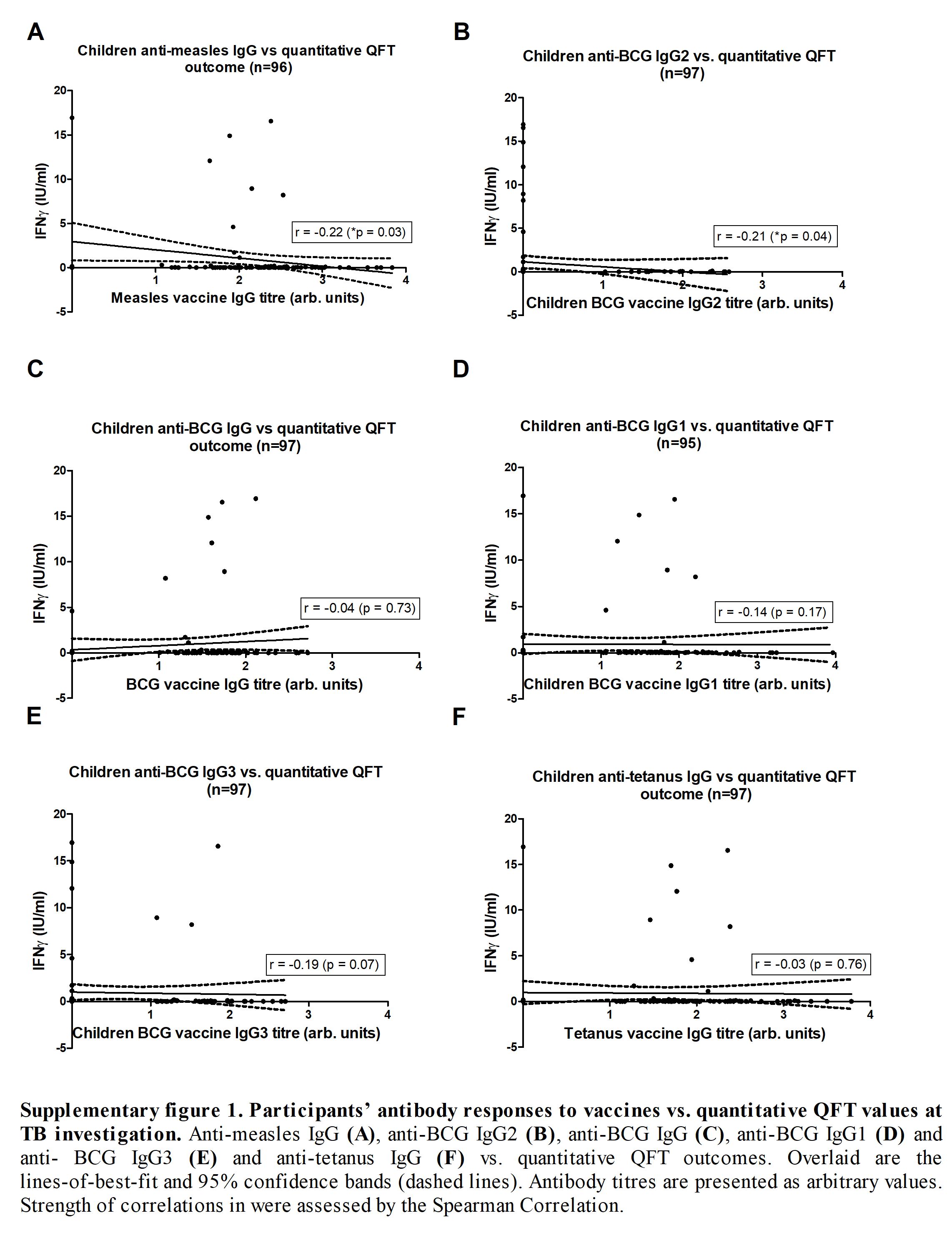

Supplement: Supplementary file 1 [file image_1.tif]

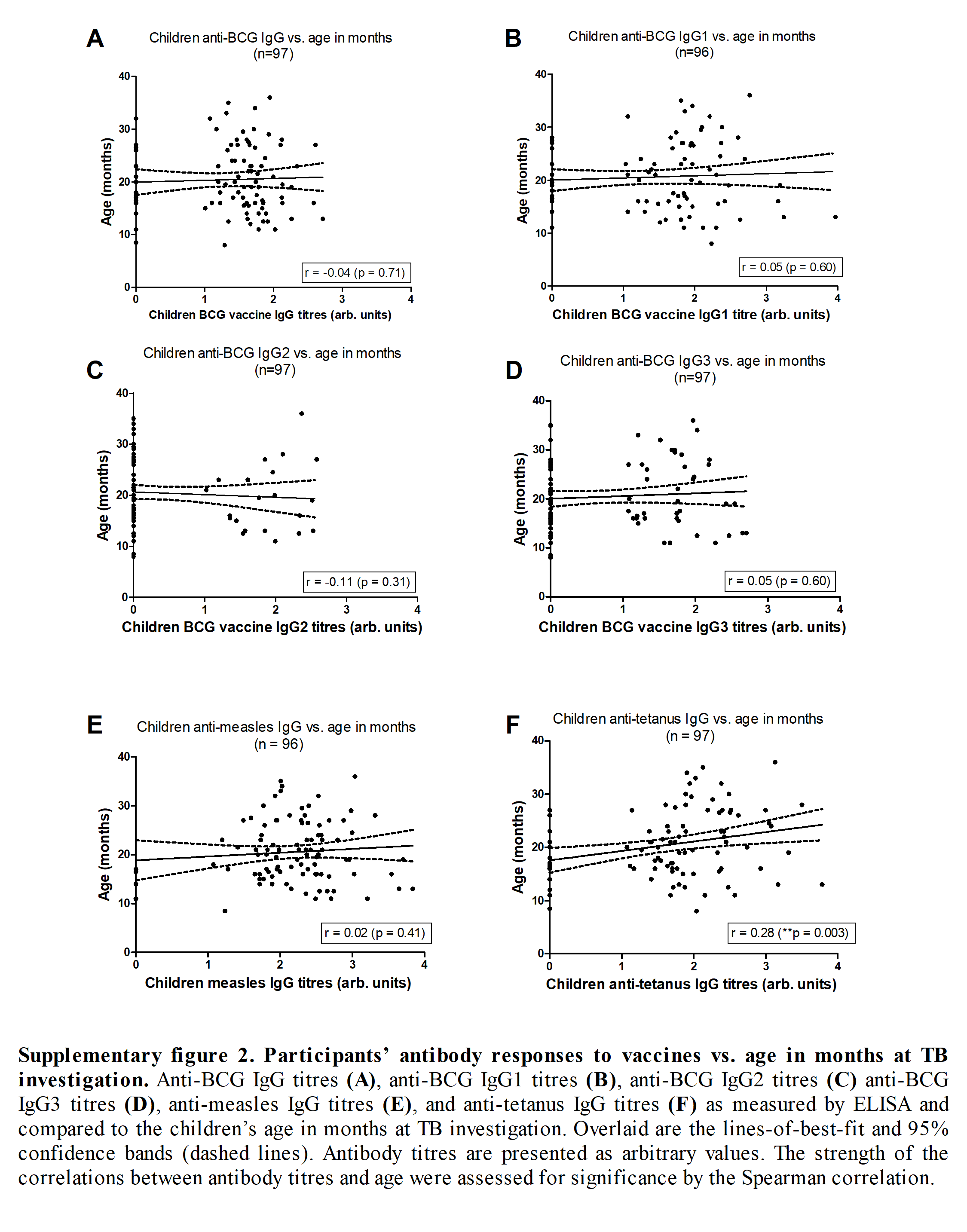

Supplement: Supplementary file 2 [file image_2.tif]

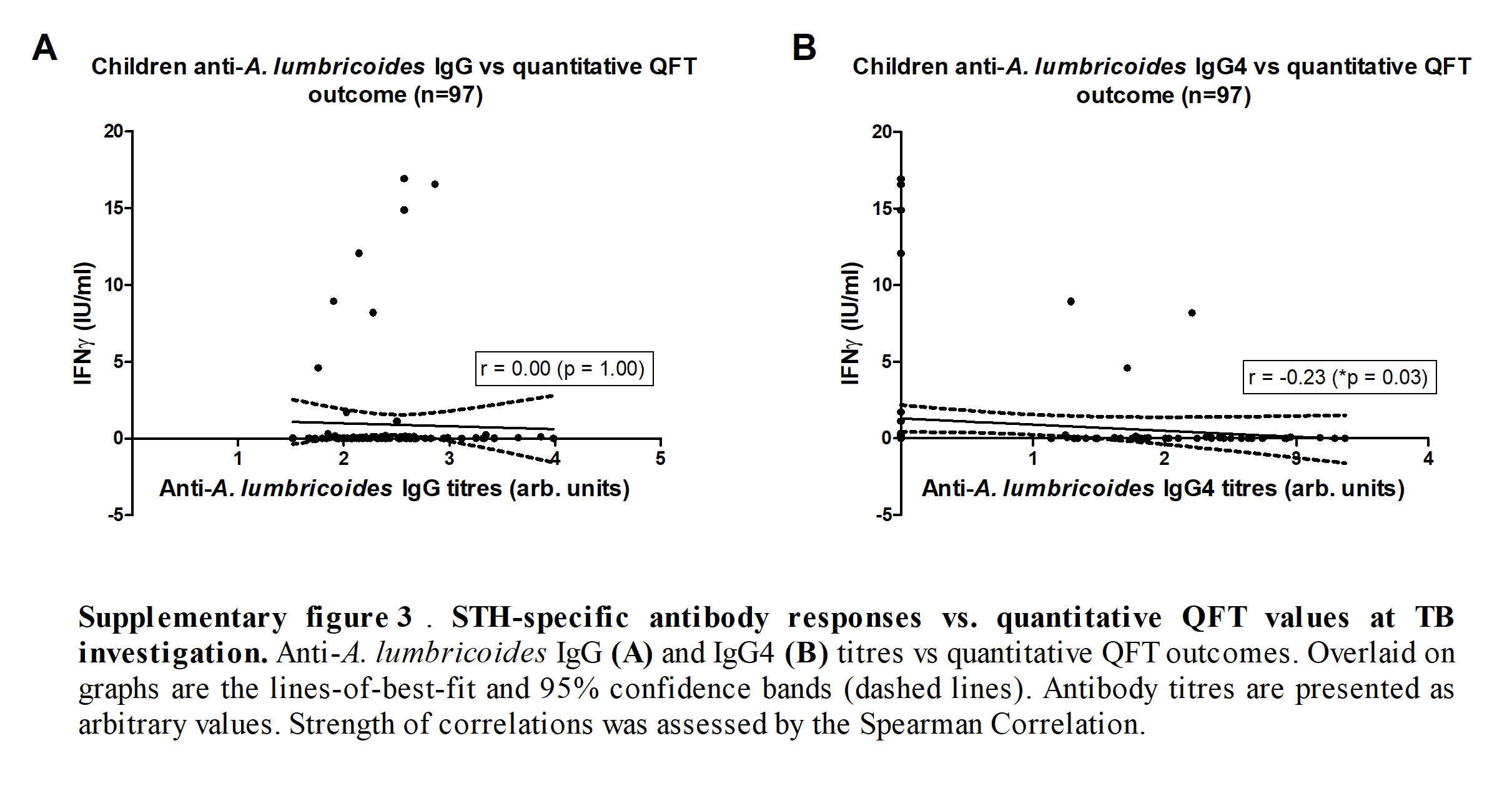

Supplement: Supplementary file 3 [file image_3.tif]
