# Supplementary material for: Elevated IgG Responses in Infants Are Associated With Reduced Prevalence of Mycobacterium tuberculosis Infection
Source: Front Immunol. 2018 Jul 2;9:1529. doi: 10.3389/fimmu.2018.01529 (PMC6036805; doi:10.3389/fimmu.2018.01529)
Supplement: Supplementary file 4 [file table_1.PDF]

Supplementary table 1. Infant housing, sanitation and helminth infection at TB investigation

	N=91
Infant characteristics	
**Housing type**	
**Flat**	4 (4.40%)
**House**	64 (70.33%)
**Informal settlement**	22 (24.18%)
**Located on farm (YES)**	34 (37.36%)
**Source of drinking water**	
**Communal**	1 (1.1%)
**Tap water**	90 (98.90%)
**Sanitation type**	
**Bucket system**	6 (6.59%)
**Flush toilet**	82 (90.11%)
**Pit latrine**	3 (3.30%)
**Prevalent helminth species**	
**At least one stool sample collected**	91 (100%)
Ascaris lumbricoides	0
Trichuris trichiura	0
**Other**	0
